# Proteins with Altered Levels in Plasma from Glioblastoma Patients as Revealed by iTRAQ-Based Quantitative Proteomic Analysis

**DOI:** 10.1371/journal.pone.0046153

**Published:** 2012-09-28

**Authors:** Poonam Gautam, Sudha C. Nair, Manoj Kumar Gupta, Rakesh Sharma, Ravindra Varma Polisetty, Megha S. Uppin, Challa Sundaram, Aneel K. Puligopu, Praveen Ankathi, Aniruddh K. Purohit, Giriraj R. Chandak, H. C. Harsha, Ravi Sirdeshmukh

**Affiliations:** 1 Centre for Cellular and Molecular Biology (CSIR), Hyderabad, India; 2 Institute of Bioinformatics, Bangalore, India; 3 Nizam’s Institute of Medical Sciences, Hyderabad, India; Baylor College of Medicine, United States of America

## Abstract

Glioblastomas (GBMs) are the most common and lethal primary tumors of the central nervous system with high level of recurrence despite aggressive therapy. Tumor-associated proteins/peptides may appear in the plasma of these patients as a result of disruption of the blood-brain barrier in them, raising the scope for development of plasma-based tests for diagnosis and monitoring the disease. With this objective, we analyzed the levels of proteins present in the plasma from GBM patients using an iTRAQ based LC-MS/MS approach. Analysis with pooled plasma specimens from the patient and healthy control samples revealed high confidence identification of 296 proteins, of which 61 exhibited a fold-change ≥1.5 in the patient group. Forty-eight of them contained signal sequence. A majority have been reported in the differentially expressed transcript or protein profile of GBM tissues; 6 have been previously studied as plasma biomarkers for GBM and 16 for other types of cancers. Altered levels of three representative proteins–ferritin light chain (FTL), S100A9, and carnosinase 1 (CNDP1)–were verified by ELISA in a test set of ten individual plasma specimens. FTL is an inflammation marker also implicated in cancer, S100A9 is an important member of the Ca^2+^ signaling cascade reported to be altered in GBM tissue, and CNDP1 has been reported for its role in the regulation of the levels of carnosine, implicated as a potential drug for GBM. These and other proteins in the dataset may form useful starting points for further clinical investigations for the development of plasma-based biomarker panels for GBM.

## Introduction

Glioblastoma (GBM) is the most common form of primary brain tumor with high mortality. Even with improved treatment modalities, the median survival of these patients is only about 15 months [1], [2]. It has been reported that the blood-brain barrier in GBM patients is compromised [3], due to which proteins/peptides from the tumor tissue may appear in the blood. The proteins that are associated with these tumors and detectable in plasma would therefore be useful to develop assays for non-invasive diagnosis or post-treatment monitoring of these patients. Various individual serum biomarkers for GBM have been reported earlier such as chitinase-3-like protein 1 (YKL-40), glial fibrillary acidic protein (GFAP), matrix metalloproteinase-9 (MMP-9), epidermal growth factor receptor (EGFR) and CD14 [4]–[8]. Reyens *et al* reported elevation of several inflammatory proteins, coagulation factors and angiogenesis factors in the plasma of GBM patients [9]. Using a combination of 2-DE/MS approach, Kumar *et al* observed ten differentially expressed proteins in the sera of patients with GBM and validated haptoglobin α2 as serum marker associated with tumor growth and migration in GBM [10]. In another study based on SELDI-TOF mass spectrometry, altered expression of alpha-chain of 2-Heremans-Schmid glycoprotein (AHSG) was shown to be correlated with prediction of survival of GBM patients [11].

We have previously used quantitative proteomics approach based on LC-MS/MS and iTRAQ to study differentially expressed membrane proteins in GBM [12]. Here, we report LC-MS/MS analysis of the plasma from GBM patients, which revealed proteins of important biological roles, including 11 of the differential membrane proteins identified in our own study referred above. Plasma analysis is an analytical challenge. Our present findings demonstrate release of tumor-associated differential proteins in the plasma of GBM patients and form the basis for clinical investigations of these proteins as well as expansion to further deeper proteome analysis of the plasma from GBM patients.

## Materials and Methods

### Plasma Samples

The Human Ethics Committee at Nizam’s Institute of Medical Sciences, Hyderabad, India had approved the study and all the blood samples were collected with written informed consent from the patients and healthy individuals at Nizam’s Institute of Medical Sciences, Hyderabad, India. Histopathological evaluation of the tumor resections was performed as per WHO guidelines. All tumors showed characteristic morphology of glioblastoma with frequent atypical mitosis, necrosis and microvascular proliferation. Blood samples from these GBM patients were collected before the surgery and administration of any medication. Samples were centrifuged at 1500× g for 20 min to obtain clear plasma and stored at -80°C until further use. The whole process was completed within 30 min after collection. Blood samples from healthy individuals, with no report of any cancer, were used as controls and processed in identical way.

Our experimental approach was to investigate differential levels of proteins in the pooled plasma samples from GBM patients as compared to the healthy controls and then to verify the mass spectrometry results in individual samples by alternative assays. Pooled GBM or control plasma samples (50–60 yr; 2 males, 1 female each) were depleted of the 14 most abundant proteins using Human 14 MARS column (4.6×100 mm; Agilent Technologies, Santa Clara, CA, USA) as per manufacturer’s instructions. Flow-through fraction was collected and desalted using mRP column (Agilent Technologies, Santa Clara, CA, USA) as per the manufacturer’s instruction and lyophilized. The samples were reconstituted in 0.1% SDS and the protein amount was estimated using Bradford’s method. The depletion was visualized by running the samples on SDS-PAGE (Figure S1). The pooled plasma samples thus prepared were used for MS analysis as described below.

### iTRAQ Labeling and SCX Fractionation

Labeling of samples with iTRAQ reagents was carried out according to the manufacturer’s instructions (iTRAQ Reagents Multiplex kit; Applied Biosystems/MDS Sciex, Foster City, CA). Briefly, 80 µg of pooled control or GBM plasma protein sample was reconstituted in dissolution buffer, denatured, reduced, alkylated and then trypsinized (4 µg modified sequencing grade trypsin; Promega, Madison, WI, USA) for 16 h at 37°C. Tryptic digests were labeled with four different iTRAQ reagents. Control samples were labeled with 114 and 115 while GBM samples with 116 and 117 iTRAQ reagents. Reactions were quenched with glycine (10 mM). All the four-labeled samples were pooled, vacuum-dried and subjected to strong cation exchange (SCX) fractionation as described earlier [12]. Eight fractions were collected and subjected to LC-MS/MS analysis.

### LC-MS/MS Analysis

Nanoflow electrospray ionization tandem mass spectrometric analysis of peptide samples was carried out using LTQ-Orbitrap Velos (Thermo Scientific, Bremen, Germany) interfaced with Agilent’s 1200 Series nanoflow LC system. The chromatographic capillary columns used were packed with Magic C_18_ AQ (particle size 5 µm, pore size 100Å; Michrom Bioresources, Auburn, CA, USA) reversed phase material in 100% ACN at a pressure of 1000 psi. The peptide sample from each SCX fraction was enriched using a trap column (75 µm × 2 cm) at a flow rate of 3 µl/min and separated on an analytical column (75 µm × 10 cm) at a flow rate of 350 nl/min. The peptides were eluted using a linear gradient of 7–30% ACN over 65 min. Mass spectrometric analysis was carried out in a data dependent manner with full scans acquired using the Orbitrap mass analyzer at a mass resolution of 60,000 at 400 m/z. For each MS cycle, twenty most intense precursor ions from a survey scan were selected for MS/MS and fragmentation detected at a mass resolution of 15,000 at m/z 400. The fragmentation was carried out using higher-energy collision dissociation (HCD) as the activation method with 40% normalized collision energy. The ions selected for fragmentation were excluded for 30 sec. The automatic gain control for full FT MS was set to 1 million ions and for FT MS/MS was set to 0.1 million ions with a maximum time of accumulation of 500 ms, respectively. For accurate mass measurements, the lock mass option was enabled.

### Protein Identification and Quantitation

The MS and MS/MS data was searched on Proteome Discoverer (Thermo Fisher Scientific, Beta Version 1.2.0.208) based on the workflow with spectrum selector and reporter ion quantifier. MS/MS search was carried out using Sequest search algorithm, against the NCBI human RefSeq database (release 40) containing 31,811 proteins. Search parameters included trypsin as the enzyme with 1 missed cleavage allowed; oxidation of methionine was set as a dynamic modification while alkylation at cysteine and iTRAQ modification at N-terminus of the peptide and lysine were set as static modifications. Precursor and fragment mass tolerance were set to 20 ppm and 0.1 Da, respectively. The peptide and protein data were extracted using high peptide confidence and top one peptide rank filters. The false discovery rate (FDR) was calculated by enabling the peptide sequence analysis using a decoy database. High confidence peptide identifications were obtained by setting a target FDR threshold of 1% at the peptide level. Relative quantitation of proteins was carried out based on the relative intensities of reporter ions released during MS/MS fragmentation of peptides. Relative intensities of the two reporter ions for each of the peptide identifiers for a protein were used for averaging and assessing percentage variability to determine relative quantity of a protein in GBM plasma sample. Only unique peptides for each protein identified were used to determine relative protein content in the two samples.

Bioinformatic analysis and annotations of the proteins identified were carried out based on their biological functions and cellular localization as per Human Protein Reference Database (HPRD, http://www.hprd.org), which is in compliance with gene ontology (GO) standards. Pathway grouping was done using the Ingenuity Pathway Knowledge Base (Ingenuity Systems, Redwood City, CA).

### Verification Assays by ELISA

Plasma levels of human ferritin light chain (FTL), S100A9 and carnosinase 1 (CNDP1) were measured in individual GBM or control plasma samples (10 subjects from each group; age range 30–60 yr; 7 males, 3 females) using ELISA quantitation kit (USCN life sciences, Wuhan, China). Fold changes in log_2_ transformed ratio for FTL, S100A9 and CNDP1 were represented using scatter plot.

## Results and Discussion

Glioblastomas are generally diagnosed on the basis of clinical evaluation, imaging and histopathological assessment of surgical biopsies. Identification of differentially expressed proteins in the plasma of GBM patients would be important to develop assays as diagnostic methods and for post treatment surveillance as viable alternatives to imaging. A number of reports are available which discuss molecules having strong potential as plasma biomarkers for GBM. YKL-40, also known as chitinase-3-like protein 1, is an extracellular matrix glycoprotein and has been reported as prognostic marker for high-grade gliomas including GBM [4]. GFAP, a major intermediate filament protein and a known astrocyte marker, has been detected to be significantly elevated in the plasma of GBM patients [5]. We have been studying GBM to understand differentially expressed proteins that are biologically relevant to the tumor state. During this effort, we identified a number of membrane-associated proteins belonging to important regulatory pathways including proteins that have secretory potential. Our independent study of the plasma from GBM patients also revealed many important proteins with altered levels. All these proteins observed provide important leads for development of clinical applications.

**Figure 1:**
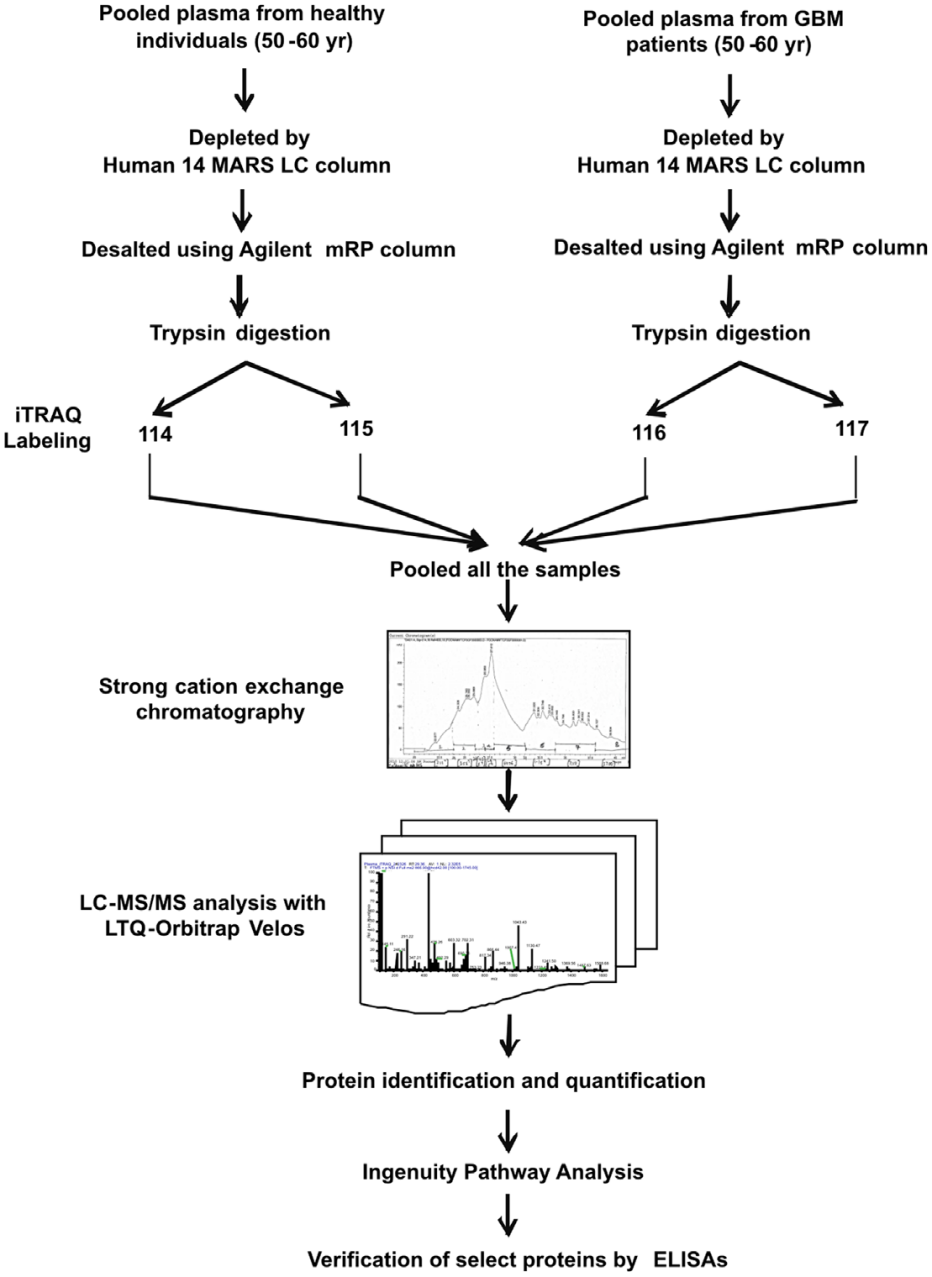
Workflow used to study differential levels of proteins in plasma from GBM patients using iTRAQ technology.

**Figure 2:**
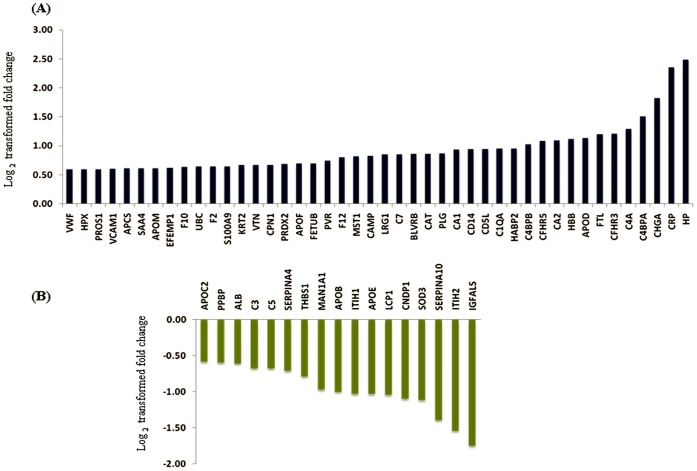
Log_2_ transformed fold changes for the proteins observed with differential levels in the plasma from GBM patients. Panel A represents up regulated proteins and Panel B down regulated proteins.

**Figure 3:**
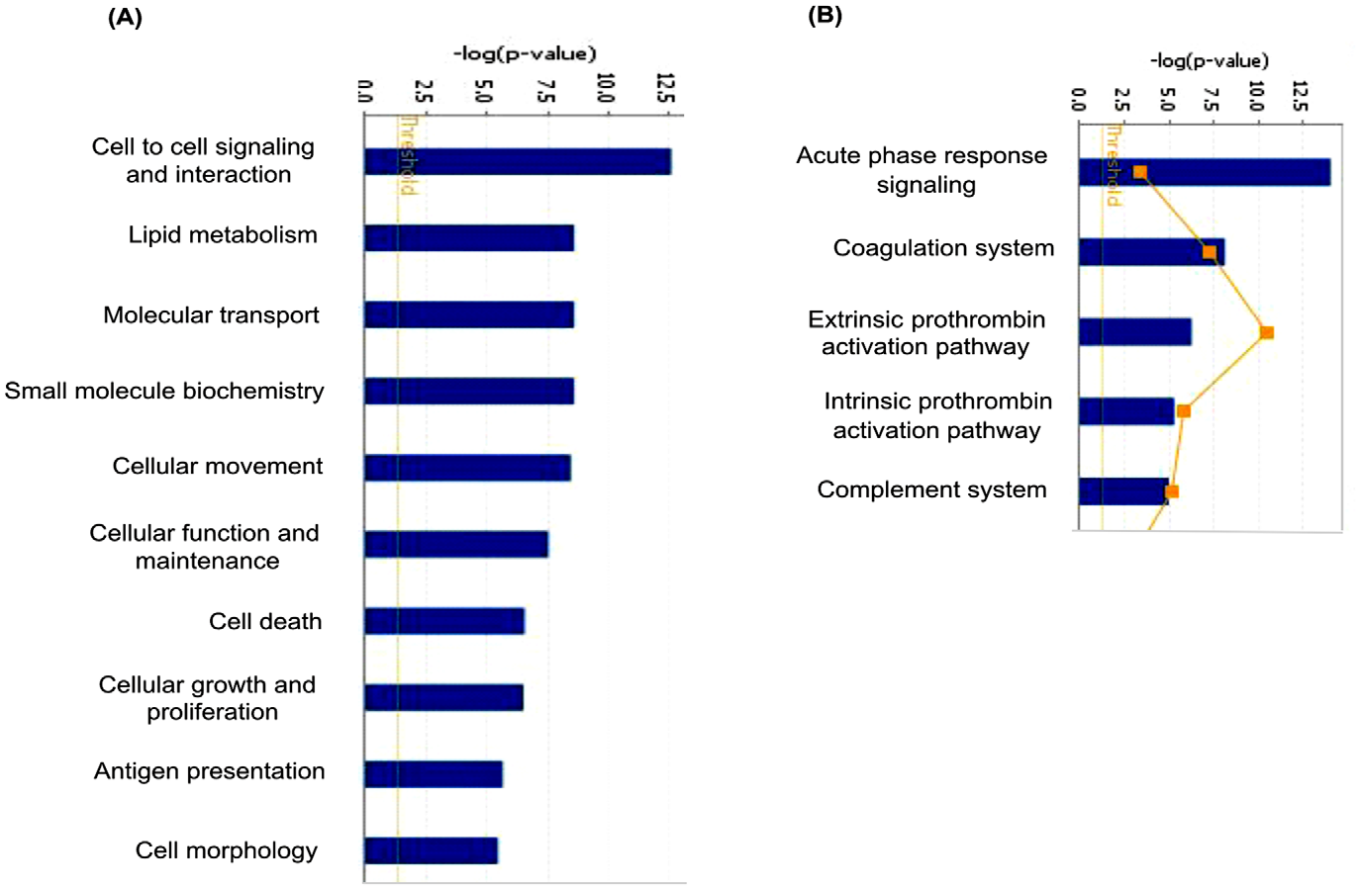
Mapping of 61 differentially regulated proteins to molecular and cellular processes and canonical pathways using Ingenuity Pathway Analysis. Top 10 cellular processes (A) and top 5 canonical pathways (B) are shown in the figure.

In our experimental approach, we compared the plasma specimens pooled from patients diagnosed with GBM samples derived from matched healthy individuals. Representative altered and functionally significant proteins were then tested in individual plasma specimens by ELISA. The pooled plasma specimens were processed for quantitative LC-MS/MS analysis using iTRAQ as summarized in Figure 1. A total of 12,976 peptides were identified which mapped to 296 proteins. These protein identifications along with their peptide information, molecular functions, biological processes and subcellular localization are provided in Table S1. A total of 61 proteins were noted with a fold change ≥1.5 and identified with at least two peptides each. The peptide representation for these differential proteins is shown in Figure S2. More than 50% of the proteins are represented by >5 peptides each. Forty-four proteins were up regulated while seventeen proteins were down regulated and the differential levels of each of them are shown in Figure 2. The altered levels of virtually all of these proteins (n = 57) correlated with the differentially expressed transcriptome data from GBM tissues [13], supporting their validity, 24 of them are reported to be altered even at the protein level in GBM tissues (Table 1). Of the 61 proteins, 51 proteins are extracellular proteins, 48 contained signal sequence indicating their membrane association and potential for secretion. Fourty-six (n = 46) proteins have been already detected in (normal) cerebrospinal fluid or plasma (Table 1). These 61 proteins could be mapped to major cellular processes such as cell-to-cell signaling and interaction, lipid metabolism, molecular transport, cellular movement, cell death and cellular growth and proliferation, on Ingenuity Pathway Analysis (IPA) analysis (Figure 3, Table S2). Top network include molecules associated with cancer, cell-to-cell signaling and interaction (Table S3A). Canonical pathways enriched were acute phase response, blood coagulation system and extrinsic prothrombin activation pathway (Table S3B).

**Table 1:** Annotations of altered plasma proteins identified in the study [35]–[52].

Gene Symbol	Protein	Peptides	Fold change	NormalCSF/Plasma	Signal/TM	Expression in tissue(Protein/mRNA)	Locali-zation
	**Upregulated proteins**						
APOD	Apolipoprotein D precursor	3	2.2	*/+	+	+	E
APOF	Apolipoprotein F precursor	2	1.6	*/+	+	+	E
APOM	Apolipoprotein M	5	1.5	*/+	+	+	E
CRP	C-reactive protein, pentraxin-related precursor	3	5.1	*/+	+	+	E
CA1	Carbonic anhydrase 1	5	1.9	*/+	–	+	C
CA2	Carbonic anhydrase 2	4	2.1	*/+	–	*/+	E
CPN1	Carboxypeptidase N, polypeptide 1 precursor	7	1.6	*/+	+	+	E
CAT	Catalase	4	1.8	*/+	–	+	C
CAMP	Cathelicidin antimicrobial peptide	3	1.8	−/+	+	*/+	E
CD14	CD14 antigen precursor	10	1.9	*/+	+	*/+	E
CD5L	CD5 antigen-like precursor	3	1.9	*/+	+	+	E
CHGA	Chromogranin A precursor	2	3.5	*/+	+	+	E
F10	Coagulation factor X preproprotein	9	1.6	*/+	+	+	E
F12	Coagulation factor XII precursor	13	1.7	*/+	+	+	E
C1QA	Complement component 1, q subcomponent, A chain precursor	3	1.9	*/+	+	+	E
C4BPA	Complement component 4 binding protein, alpha chain precursor	15	2.8	*/+	+	+	E
C4BPB	Complement component 4 binding protein, beta chain isoform 2 precursor	7	2.0	−/+	+	+	E
C4A	Complement component 4A preproprotein	12	2.5	*/+	+	*/−	E
C7	Complement component 7 precursor	23	1.8	*/+	+	+	E
CFHR3	Complement factor H-related 3 isoform 1 precursor	2	2.3	*/+	+	–	E
CFHR5	Complement factor H-related 5 precursor	6	2.1	−/+	+	*/+	E
EFEMP1	EGF-containing fibulin-like extracellular matrix protein 1 precursor	5	1.5	*/+	+	*/+	E
FTL	Ferritin, light polypeptide	4	2.3	*/+	–	*/+	C
FETUB	Fetuin-B precursor	7	1.6	*/+	+	+	E
BLVRB	Flavin reductase	5	1.8	−/+	–	+	C
HP	Haptoglobin isoform 2 preproprotein	2	5.6	*/+	+	*/+	E
HBB	Hemoglobin subunit beta	6	2.2	*/+	–	*/+	E
HPX	Hemopexin precursor	28	1.5	*/+	+	*/+	E
HABP2	Hyaluronan binding protein 2 preproprotein	7	1.9	*/+	+	+	E
KRT2	Keratin 2	5	1.6	*/+	–	*/+	C
LRG1	Leucine-rich alpha-2-glycoprotein 1 precursor	12	1.8	*/+	+	+	E
MST1	Macrophage stimulating 1 precursor	11	1.8	*/+	+	*/+	E
PRDX2	Peroxiredoxin-2 isoform a	5	1.6	*/+	–	*/+	C
PLG	Plasminogen isoform 1 precursor	43	1.8	*/+	+	+	E
PVR	Poliovirus receptor isoform gamma	2	1.7	*/+	+	*/+	E
PROS1	Protein S, alpha preproprotein	10	1.5	*/+	+	+	E
S100A9	Protein S100-A9	4	1.6	*/+		*/+	E
F2	Prothrombin preproprotein	35	1.6	*/+	+	*/+	E
SAA4	Serum amyloid A-4 protein precursor	2	1.5	*/+	+	+	E
APCS	Serum amyloid P component precursor	6	1.5	*/+	+	+	E
UBC	Ubiquitin C	3	1.6	−/+		+	C
VCAM1	Vascular cell adhesion protein 1 isoform a precursor	3	1.5	*/+	+	*/+	E
VTN	Vitronectin precursor	9	1.6	*/+	+	*/+	E
VWF	Von Willebrand factor preproprotein	3	1.5	*/+	+	*/+	E
	**Downregulated proteins**						
ALB	Albumin preproprotein	8	0.7	*/+	+	*/+	E
APOB	Apolipoprotein B precursor	78	0.5	*/+	+	+	E
APOC2	Apolipoprotein C-II precursor	4	0.7	*/+	+	+	E
APOE	Apolipoprotein E precursor	13	0.5	*/+	+	*/+	E
CNDP1	Carnosinase 1 precursor	9	0.5	*/+	+	–	C
C3	Complement component 3 precursor	6	0.63	*/+	+	*/−	E
C5	Complement component 5 preproprotein	40	0.63	*/−	+	+	E
IGFALS	Insulin-like growth factor binding protein, acid labile subunit isoform 2 precursor	15	0.3	*/+	+	+	E
ITIH1	Inter-alpha (globulin) inhibitor H1 isoform a	20	0.5	*/+	+	+	E
ITIH2	Inter-alpha globulin inhibitor H2 polypeptide	24	0.3	*/+	+	*/+	E
MAN1A1	Mannosyl-oligosaccharide 1,2-alpha-mannosidase IA	3	0.5	*/+	+	+	ER
LCP1	Plastin-2	2	0.5	*/+		+	C
PPBP	Pro-platelet basic protein precursor	5	0.7	*/+	+	*/+	E
SERPINA10	Serine proteinase inhibitor, clade A, member 10 precursor	6	0.4	*/+	+	+	E
SERPINA4	Serine proteinase inhibitor, clade A, member 4 precursor	15	0.6	*/+	+	+	E
SOD3	Superoxide dismutase 3, extracellular precursor	2	0.5	*/+	+	+	E
THBS1	Thrombospondin 1 precursor	16	0.6	*/+	+	+	E

Footnote:

E- Extracellular; C- Cytoplasm; ER- Endoplasmic reticulum.

In column 5 (*) indicates the earlier report of a protein identified in normal CSF and (+) in normal Plasma.

In column 7 (*) indicates expression of a protein in tissue at protein level (+) at mRNA level.

Protein localization, Signal/TM domain containing information was derived from HPRD [35] and information about presence in normal CSF or plasma was extracted from [36], [37] respectively. Expression of the genes/proteins at tissue level was inferred from published transcriptome dataset (master list; [38]) or protein datasets [39]–[52].

Acute phase reactant proteins (APRPs) are associated in various types of cancers as well as other clinical conditions. These may be a result of inflammatory responses. Major APRPs observed are C-reactive protein (CRP) and haptoglobin α2, which were previously reported to be elevated in the plasma of patients with GBM [9], [10]. The present study reports increased levels of additional APRPs such as plasminogen and coagulation factor XII which suggest the release of active plasmin acting as a proteolytic factor in inflammatory reactions and tumor invasion [14]. Other APRPs such as ferritin light chain, hemopexin and serum amyloid A-4,observed by us, are implicated in other cancers as well [15]–[18]. Along with these APRPs, another striking protein observed to be altered was S100A9, a member of calcium signaling pathway. Both these groups, including FTL and S100A9 were also revealed in the analysis with GBM tissues. The signal sequence containing proteins, C-reactive protein, CD14 antigen precursor, haptoglobin α2, vascular cell adhesion protein 1, serum albumin and thrombospondin-1 identified in our analysis, have also been reported earlier in the plasma of GBM patients [8], [10], [19], while others (n = 42), observed here are being reported for the first time. Chromogranin (CgA) is an acidic glycoprotein commonly overexpressed in neuroendocrine tumors [20] and viewed as a biomarker for the diagnosis of neuroendocrine tumors [21], [22]. Endothelial carbonic anhydrase, CA II, was earlier shown to be associated with a poor prognosis in astrocytoma patients [23]. Fibulin 3 (EFEMP1) promotes tumor cell invasion and mobility [24]. Another protein Carnosinase 1 (CNDP1) is a brain-associated protein. Serum carnosinase (CNDP1) is synthesized in the brain and secreted into the cerebrospinal fluid and then into the blood. Its deficiency has been associated with various neurologic deficits [25]. The protein may have therapeutic role (see discussion below).

**Figure 4:**
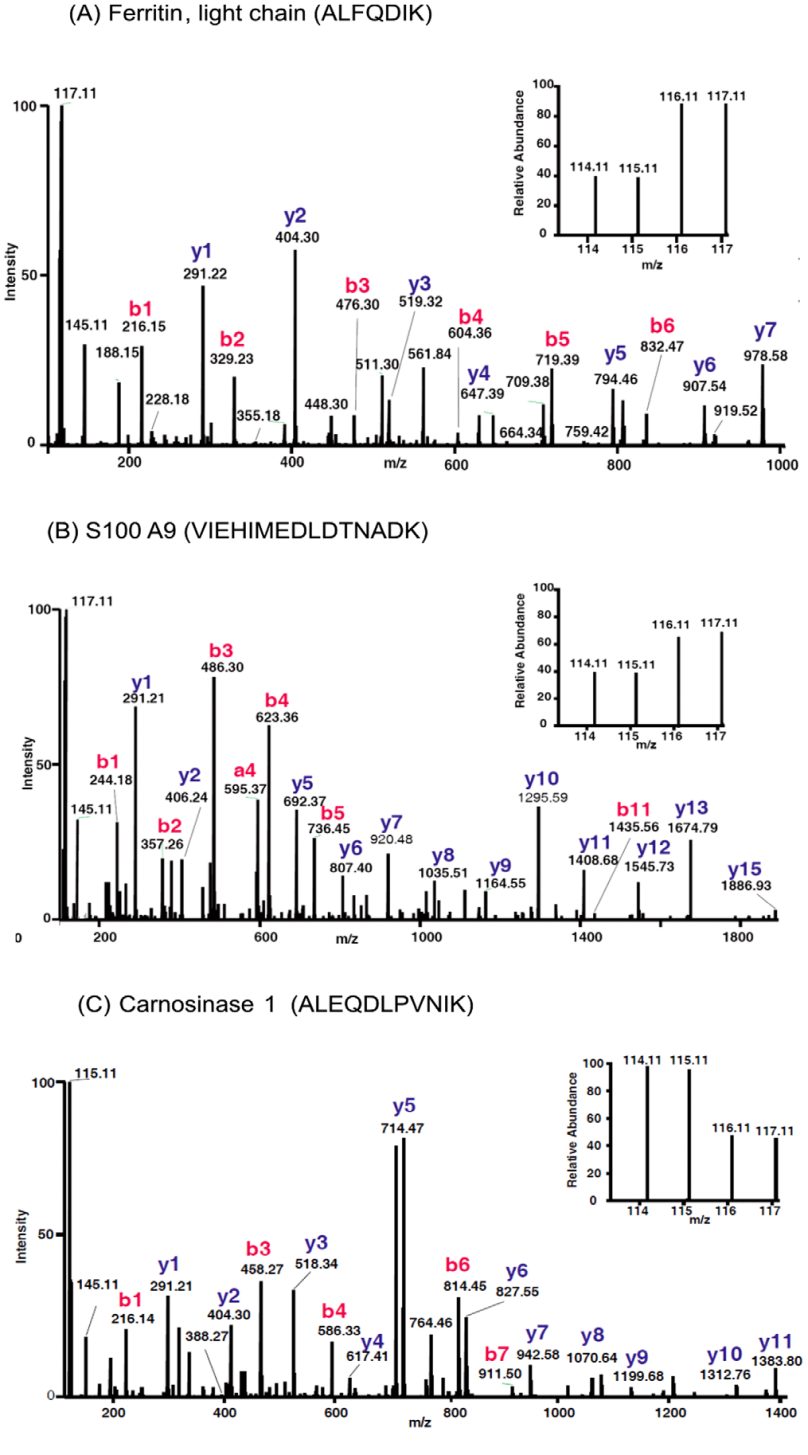
MS/MS spectra of select peptides with their reporter ions for three proteins - ferritin light chain, S100A9 and carnosinase 1. In the analysis, iTRAQ reporter ions114 and 115 represent control specimens whereas 116 and 117 represent plasma from GBM patients.

**Figure 5:**
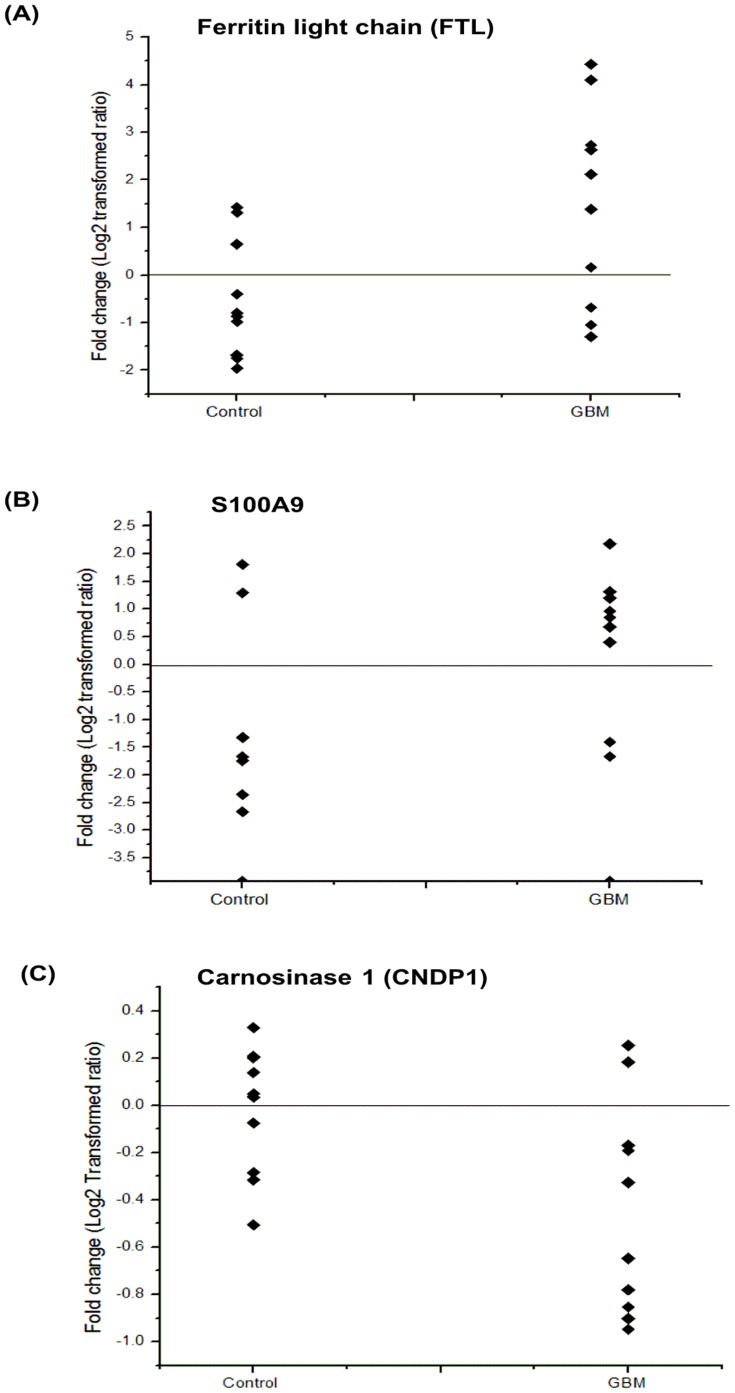
Scatter plot representing altered levels of ferritin light chain, S100A9 and carnosinase 1, in individual specimens from control subjects and GBM patients as determined by ELISA. Elevated levels of Ferritin light chain were observed in 7 out of 10 GBM cases and in 8 out of 10 patients for S100A9. Lower levels of Carnosinase 1were observed in 8 out of 10 GBM patients. The fold changes are shown in log_2_ transformed ratio. The details of ELISA are given under Methods.

We selected and further examined the level of three proteins FTL, S100A9 and CNDP1. The MS/MS spectra of the representative peptides of these three proteins along with their reporter ions, obtained for the plasma samples are shown in Figure 4. Consistent to the results of iTRAQ analysis, we observed significantly elevated levels of FTL and S100A9 and reduced levels of CNDP1 in individual plasma specimens from GBM patients. The fold changes of these proteins, as log_2_ transformed ratio, are shown in the scatter plot (Figure 5). Elevated levels of FTL were observed in 7 out of 10 GBM cases whereas for S100A9 in 8 out of 10 patients. Reduced levels of CNDP1 were observed in 8 out of 10 plasma specimens. Ferritin is an acute phase protein and involved in iron storage. Almost all cells possess the ability to synthesize ferritin, including glial cells, in the central nervous system [26]. Serum ferritin, a glycosylated protein composed primarily of L-subunit type, has been reported to be elevated in various cancers including stomach and head and neck cancers [15], [27]. It is also shown to be elevated in cerebrospinal fluid of GBM patients [28] suggesting its secretion by glial cells. Since, serum ferritin is also reported in other inflammatory conditions and it may simply represent tumor related inflammatory environment. S100A9 is a calcium-binding protein also likely to be contributed by the inflammatory cells in the tumor microenvironment and is observed to be elevated in the tumors. S100A9 is co-expressed with S100A8 to form a functional complex and is critically involved in tumor- stroma interactions. It is mainly localized in the cytosol but translocates to membrane upon elevated intracellular calcium levels [29]. As secreted factors, they are involved in the recruitment of tumor cells into ‘pre-metastatic niches’ [30]. We did detect altered levels of S100A8 in the tumor tissue [12], although not in the plasma. Consistently observed altered levels of FTL and S100 A9 in the plasma from GBM patients qualifies them for further investigation for their clinical applications. CNDP1 is involved in the metabolism of carnosine and carnosine homeostasis is implicated in multiple functions and any disturbance in it may have crucial metabolic consequences [31]. Carnosine is also reported to have anti-growth property and has been discussed for its therapeutic potential against tumors including GBM [32]. Carnosinase, which hydrolyzes carnosine, exists in two distinct isoforms and has been discussed in the context of major neurodegenerative condition [33]. The serum isoform, CNDP1, is distinct and our identification is supported by the unique peptides for this form. Reduced levels of CNDP1 have been observed in patients with Parkinson disease or multiple sclerosis and in patients after a cerebrovascular accident [34]. Our analysis, for the first time, shows that serum CNDP1 is present at reduced levels in the plasma of GBM patients, which may be important in the maintenance of carnosine levels and bioavailability of carnosine as a drug for GBM [33].

In summary, plasma-based tests for diagnosing GBM or for its recurrence would be highly useful. Our initial, unbiased proteomics analysis of the plasma from GBM patients reveals altered proteins that are biologically important and implicated in the context of inflammatory reactions or cancer in general as well as glioblastoma in particular. FTL, S100A9 and CNDP1, have already shown encouraging reproducibility in a set of 10 individual specimens. These and others which would pass the reproducibility test will form useful panels for investigation for their diagnostic potential. Reduced levels of CNDP1 may have important therapeutic implications in the application of carnosine as a drug for GBM. Our results thus provide a valuable resource of proteins and permit further investigation on the plasma from GBM patients for extension to clinical applications.

## Supporting Information

Figure S1 **SDS-PAGE analysis of Ag14 depleted and desalted pooled plasma proteins from control and GBM subjects.** 25 µg of protein was loaded on 4–20% gradient gel and stained with Coomassie brilliant blue to visualize the proteins.
(TIF)

Figure S2 **Peptide coverage for the differential proteins observed.**
(TIF)

Table S1 List of all plasma proteins (n = 296) identified in the study along with peptides, molecular function, biological process and localizations.
(XLS)

Table S2 Differentially expressed proteins associated with major molecular and cellular functions as assessed with Ingenuity Pathway Analysis (IPA). Differentially expressed proteins from Table 1 with 1.5 fold change were used for the analysis.
(DOC)

Table S3 Ingenuity Pathway Analysis of the differentially expressed plasma proteins associated with major networks and processes (A) and those associated canonical pathways (B). Differentially expressed proteins from Table 1 were used for the analysis and are shown in bold. Only top three networks or pathways are shown.
(DOC)
