# Supplementary figures and images for: Proteins with Altered Levels in Plasma from Glioblastoma Patients as Revealed by iTRAQ-Based Quantitative Proteomic Analysis
Source: PLoS One. 2012 Sep 28;7(9):e46153. doi: 10.1371/journal.pone.0046153 (PMC3461020; doi:10.1371/journal.pone.0046153)

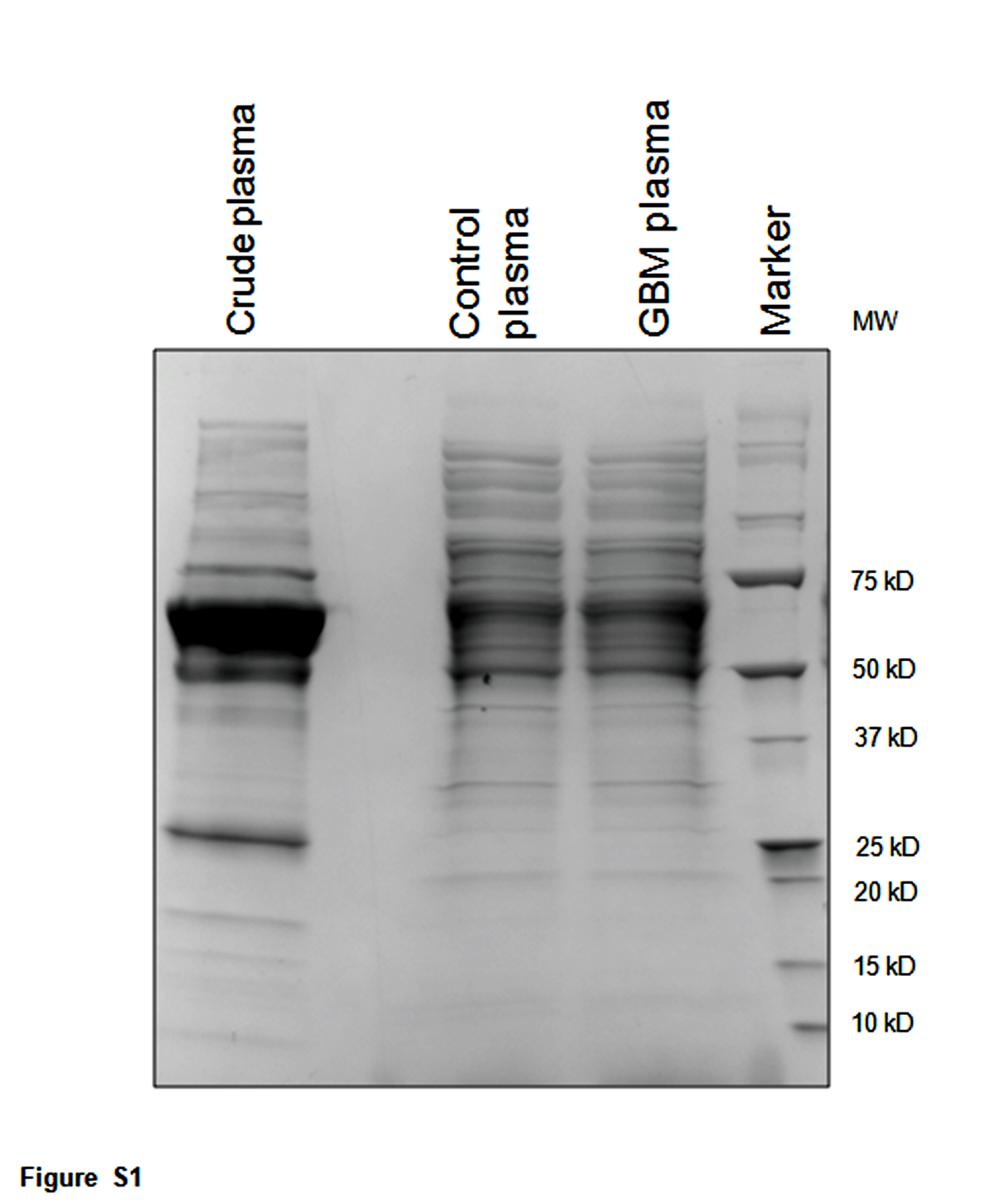

Supplement: Figure S1 — SDS-PAGE analysis of Ag14 depleted and desalted pooled plasma proteins from control and GBM subjects. 25 µg of protein was loaded on 4–20% gradient gel and stained with Coomassie brilliant blue to visualize the proteins. (TIF) [file pone.0046153.s001.tif]

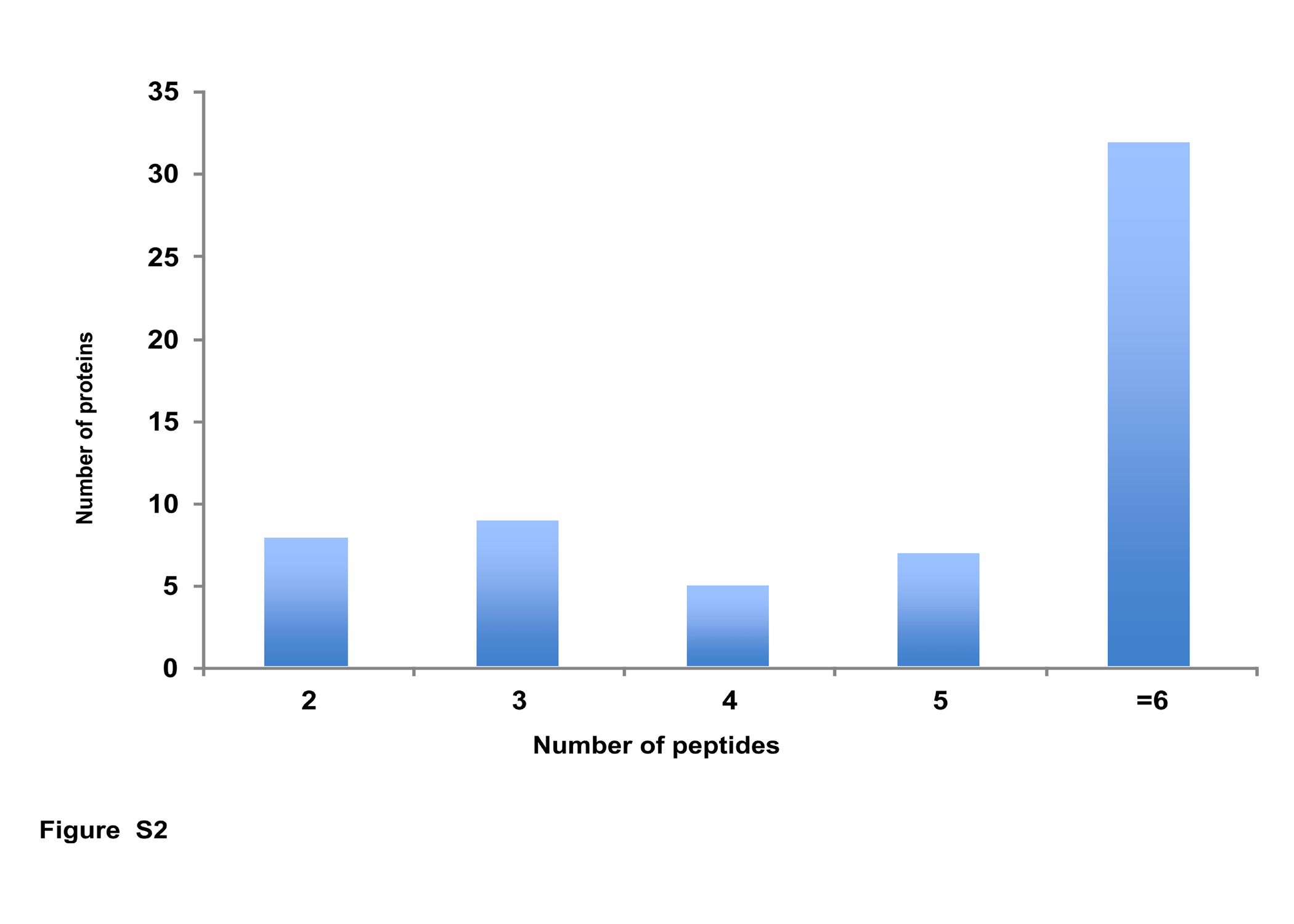

Supplement: Figure S2 — Peptide coverage for the differential proteins observed. (TIF) [file pone.0046153.s002.tif]
