# Supplementary material for: Proteins with Altered Levels in Plasma from Glioblastoma Patients as Revealed by iTRAQ-Based Quantitative Proteomic Analysis
Source: PLoS One. 2012 Sep 28;7(9):e46153. doi: 10.1371/journal.pone.0046153 (PMC3461020; doi:10.1371/journal.pone.0046153)
Supplement: Table S2 — Differentially expressed proteins associated with major molecular and cellular functions as assessed with Ingenuity Pathway Analysis (IPA). Differentially expressed proteins from Table 1 with 1.5 fold change were used for the analysis. (DOC) [file pone.0046153.s004.doc]

**Table S2**

**Differentially expressed proteins associated with major molecular and cellular functions as assessed with Ingenuity Pathway Analysis (IPA).** Differentially expressed proteins from Table 1 with 1.5 fold change were used for the analysis.

| **Category** | **Molecules** | ***P*-value** |
| --- | --- | --- |
| Cell-To-Cell Signaling and Interaction | F12, APOE, APOA4, LPA, VTN, F2, CD5L, APCS, APOC3, PROS1, PPBP, KRT2, VCAM1, TGFBI, THBS1, VWF, PLG, F10, ALB, CAMP, S100A9, HABP2, CRP, CAT, C4B (includes others), CD14, PRDX2 | 2.62E-13-8.67E-03 |
| Lipid Metabolism | APOE, LPA, APOM, APOA4, APOB, THBS1, APOF, VTN, APOC2, F2, PLG, F10, ALB, CAMP, S100A9, APOC3, CRP, CAT, PPBP, CD14, PRDX2, APOD | 2.55E-09-7.47E-03 |
| Molecular Transport | APOE, VCAM1, APOM, LPA, APOA4, APOB, CHGA, THBS1, APOF, VWF, APOC2, F2, PLG, ALB, CAMP, S100A9, APOC3, CAT, PPBP, CRP, CD14, PRDX2, APOD | 2.55E-09-8.67E-03 |
| Small Molecule Biochemistry | APOE, APOA4, APOB, LPA, APOF, VTN, APOC2, F2, HBB, ITIH2, APOC3, PPBP, APOM, CHGA, THBS1, PLG, F10, HP, ALB, CAMP, S100A9, CRP, CAT, CD14, EFEMP1, APOD, PRDX2 | 2.55E-09-7.47E-03 |
| Cellular Movement | APOE, LCP1, VCAM1, LPA, THBS1, VTN, VWF, MST1, PVR, F2, PLG, F10, ALB, CAMP, S100A9, CRP, CAT, PPBP, PROCR, CD14, KRT2 | 3.55E-09-5.78E-03 |
| Cellular Function and Maintenance | F10, APOE, CAMP, APCS, THBS1, APOC3, PROS1, VTN, CRP, CD14, APOC2 | 2.96E-08-4.34E-03 |
| Cell Death | CA2, APOE, VCAM1, APOB, THBS1, VTN, MST1, PVR, SOD3, F2, PLG, HP, ALB, HBB, CAMP, S100A9, APOC3, PPBP, CAT, CRP, PROCR, CD14, UBC, PRDX2 | 2.81E-07-8.67E-03 |
| Cellular Growth and Proliferation | APOE, F12, VCAM1, FTL, LPA, TGFBI, THBS1, CHGA, VTN, MST1, F2, PLG, CAMP, HABP2, CAT, PROCR, CD14, KRT2 | 3.14E-07-7.96E-03 |
| Antigen Presentation | APOE, VCAM1, LPA, THBS1, VTN, MST1, PLG, CAMP, S100A9, APCS, PROS1, CRP, PPBP, CD14 | 2.23E-06-8.67E-03 |
| Cell Morphology | PLG, ALB, VCAM1, CAMP, TGFBI, THBS1, VTN, PROCR, KRT2, MST1, PVR, F2 | 3.54E-06-4.34E-03 |
