# Supplementary material for: Proteins with Altered Levels in Plasma from Glioblastoma Patients as Revealed by iTRAQ-Based Quantitative Proteomic Analysis
Source: PLoS One. 2012 Sep 28;7(9):e46153. doi: 10.1371/journal.pone.0046153 (PMC3461020; doi:10.1371/journal.pone.0046153)
Supplement: Table S3 — Ingenuity Pathway Analysis of the differentially expressed plasma proteins associated with major networks and processes (A) and those associated canonical pathways (B). Differentially expressed proteins from Table 1 were used for the analysis and are shown in bold. Only top three networks or pathways are shown. (DOC) [file pone.0046153.s005.doc]

**Table S3 Ingenuity Pathway Analysis of the differentially expressed plasma proteins associated with major networks and processes (A) and those associated canonical pathways (B).** Differentially expressed proteins from Table 1 were used for the analysis and are shown in bold. Only top three networks or pathways are shown.

(A)

| **ID** | **Top Functions** | **Molecules in Network** | **Score** | **Focus Molecules** |
| --- | --- | --- | --- | --- |
| 1 | Cancer, Cell-To-Cell Signaling and Interaction | **ALB**, **APCS**, **APOD, C4BPA**, CD163, CDKN1A, CEBPB, **CHGA**, COL18A1, **CRP**, CTNNB1, F2**, F12**, FURIN, **HABP2**, HMOX1, HNF1A, **HP**, IL6, IL17A, IL6R, LUM, N-cor, NFYA, NR5A2, PCSK6, PLAU, RBPJ, SERPINE1, SMAD4, SPINT2, TGFB1, **THBS1, VWF** | 23 | 11 |
| 2 | Inflammatory Response, Antigen Presentation, Cell-To-Cell Signaling and Interaction | **APOB, APOC2,** APOC3**, APOE, APOM, CAT**, CCL4, CCL7, CD163, CEBPB, **CRP**, CXCL1, EPO, Ferritin, HLA-DR, HNF4A, IFNG, IKBKG, IL33, IL1B, **LCP1**, lymphotoxin-alpha1-beta2, **MST1**, MUC2, NR1H3, NR5A2, **PPBP, PRDX2**, RNASE2, S100A8, **S100A9**, SCARB1, **SOD3**, TNF, **UBC** | 23 | 13 |
| 3 | Cell-To-Cell Signaling and Interaction, Inflammatory Response, Cellular Movement | AGER, **APOB, APOE**, ARHGAP5, **C4B (includes others), CAMP, CAT, CD14,** CETP, **CRP**, CXCR2, ERK1/2, F2**, F10,** Focal adhesion kinase, FPR2, HSP90B1, IL1, IL6R, LDL, LPL, LY96, NFkB (complex), OLR1, P2RY2, **PLG, PROS1**, S1PR1, SAA, SCARB1, SERPINC1, SFTPA1, TIMP2, **VCAM1, VTN** | 21 | 13 |

**(B)**

| **Canonical Pathway** | ***P*-value** | **Ratio** | **Molecules** |
| --- | --- | --- | --- |
| Acute Phase Response Signaling | 9.48E-15 | 14/172 | **HPX, FTL, C4BPB, VWF, F2, PLG, ALB, HP, ITIH2, C4BPA, APCS, SAA4, CRP, C4B (includes others)** |
| Coagulation System | 7.73E-09 | 6/35 | **PLG, F10, F12, PROS1, VWF, F2** |
| Extrinsic Prothrombin Activation Pathway | 5.65E-07 | 4/16 | **F10, F12, PROS1, F2** |
